# Impact-related microspherules in Late Pleistocene Alaskan and Yukon “muck” deposits signify recurrent episodes of catastrophic emplacement

**DOI:** 10.1038/s41598-017-16958-2

**Published:** 2017-11-30

**Authors:** Jonathan T. Hagstrum, Richard B. Firestone, Allen West, James C. Weaver, Ted E. Bunch

**Affiliations:** 1 U.S. Geological Survey, Menlo Park, CA 94025 USA; 2 Lawrence Berkeley National Laboratory, Berkeley, CA 94720 USA; 3 Comet Research Group, Prescott, AZ 86301 USA; 4 Wyss Institute for Biologically Inspired Engineering, Harvard University, Cambridge, MA 02138 USA; 5 School of Earth Science and Environmental Sustainability, Northern Arizona University, Flagstaff, AZ 86011 USA

## Abstract

Large quantities of impact-related microspherules have been found in fine-grained sediments retained within seven out of nine, radiocarbon-dated, Late Pleistocene mammoth (*Mammuthus primigenius*) and bison (*Bison priscus*) skull fragments. The well-preserved fossils were recovered from frozen “muck” deposits (organic-rich silt) exposed within the Fairbanks and Klondike mining districts of Alaska, USA, and the Yukon Territory, Canada. In addition, elevated platinum abundances were found in sediment analysed from three out of four fossil skulls. In view of this new evidence, the mucks and their well-preserved but highly disrupted and damaged vertebrate and botanical remains are reinterpreted in part as blast deposits that resulted from several episodes of airbursts and ground/ice impacts within the northern hemisphere during Late Pleistocene time (~46–11 ka B.P.). Such a scenario might be explained by encounters with cometary debris in Earth-crossing orbits (Taurid Complex) that was generated by fragmentation of a large short-period comet within the inner Solar System.

## Introduction

**Figure 1:**
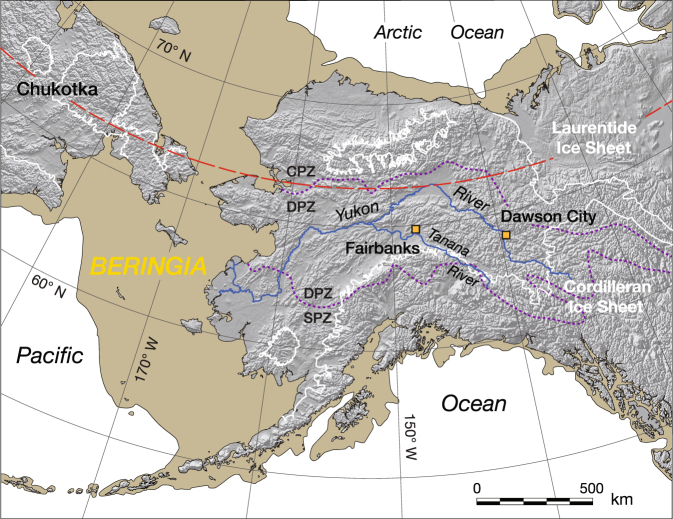
Map showing eastern Beringia during the Last Glacial Maximum (LGM; ~23–19 ka B.P.) when eustatic sea level was ~120 m below its present level^1^. Megafauna fossils of this study were initially collected between 1940 and 1954 from mining operations near Fairbanks, Alaska, and more recently from the Klondike mining district near Dawson City in the Yukon Territory. Permafrost zones, discriminated by dashed purple curves, are continuous (CPZ, >90% frozen ground), discontinuous (DPZ, >50%), and sporadic (SPZ, <50%)^35^. White curves outline areas of both continental and mountain glaciation with the latter having occurred in the Brooks Range of northern, and the Alaskan Range of southern, Alaska^70^. Dashed red curve indicates the Arctic Circle. Chukotka is an autonomous district (Okrug) in easternmost Russia. The shaded-relief base map (SR_HR.tif) was acquired from Natural Earth (http://www.naturalearthdata.com) and modified as indicated above in Esri ArcGIS Desktop, v. 10.2 (http://www.arcgis.com).

During the time period leading up to the Last Glacial Maximum (~23–19 ka B.P.), when eustatic sea level was substantially lower, Alaska and the Yukon Territory were part of the largest circumarctic area to remain unglaciated, called Beringia (Fig. 1), which extended from eastern Siberia (Chukotka) across the exposed Bering Strait region into Alaska and western Canada^1,2^. The glacial steppe environment of Beringia^3^ was a refugium for Plio-Pleistocene tundra-grassland plant communities^4^ as well as for the now-extinct mammalian megafauna^5^. Relict permafrost within the region^6–9^ has preserved an extraordinary frozen record of plant, pollen, insect, and vertebrate fossil remains, as well as their ancient DNA^1,10,11^.

Early expeditions to Alaska^12–14^ and the Yukon Territory^14,15^ found large quantities of megafaunal bones along beaches, riverbanks, and in minor-stream valleys^8^. Even greater collections of these fossils were made after industrial-scale placer-gold mining operations began in the Fairbanks and Klondike districts in the early 20^th^ century^16^. Otto W. Geist undertook extensive fossil collecting in Alaska on behalf of the American Museum of Natural History (AMNH), and in a typical year (1938) shipped more than eight thousand select specimens, weighing nearly eight tons (~7257 kg), to New York City^8^.

The fossil bones collected included those of bison, mammoth, horse, musk ox, moose, lynx, lion, camel, mastodon, bear, and caribou, with many of these animals also appearing as frozen partial carcasses or mummies^1,8,17^. The three most common genera found were bison, mammoth, and horse, which represent more than 90% of Beringia’s large mammalian biomass^18^. Many tens of thousands of specimens were collected in the 20^th^ century from Alaska and the Yukon Territory^5,8,19^, and hundreds to thousands more are still being recovered every year from mines in the Klondike district alone^1^.

Unfortunately, the stratigraphic context of most Beringian vertebrate fossils in museum collections is unknown because they were usually found already exhumed on beaches, riverbanks, or the floors of mining excavations^7,8^. Radiocarbon dates^19,20^ and those fossils recovered with stratigraphic information, however, indicate that the megafaunal remains are predominantly from Wisconsinan-aged deposits^8,9,21,22^. Furthermore, by the end of the Wisconsinan Glacial Stage (~85–11.7 ka B.P.) and Pleistocene Epoch (~2.58 Ma–11.7 ka B.P.) much of the mammalian megafauna of Eurasia and North America had become extinct. A number of causes for the Late Pleistocene extinctions have been proposed including intensive human hunting^23,24^, climate change^20,25^, and extraterrestrial impacts^26–28^, and the subject remains highly controversial^29,30^.

The demise of animals whose remains have been found in Beringian mucks has long been attributed to “natural deaths” in a “rigorous environment”^8^, and researchers have generally held to non-catastrophic or “uniformitarian concepts” in their interpretations of the causal events leading up to fossil preservation^5,31^. Uniformitarian explanations can often appear inadequate, however, in providing a clear picture of how, for instance, animals found as frozen carcasses were quickly killed, often dismembered, and buried without normal predation, scavenging, or decomposition prior to freezing^5^.

Otto Geist held a more catastrophic view, based on his observations as collector, and envisioned a rapid series of events beginning with large volcanic eruptions and dust storms depositing widespread ash and silt beds that starved the animal grazers, followed by “great winds” that stripped trees of their leaves and bark before knocking them down, and finally by floodwaters that dismembered the many carcasses while washing them along with silt and plant debris into the creek valleys^32^. No evidence of large-scale starvation or floodwaters has been found, and no sources of “great winds” have been identified, so the scientific community has largely dismissed such catastrophic interpretations.

Herein we present the discovery of impact-related microspherules and elevated platinum concentrations in fine-grained sediments retained within seven Late Pleistocene bison and mammoth skull fragments from Alaska and the Yukon Territory, which potentially indicate a catastrophic origin for at least part of the frozen Beringian mucks. Our results point to repeated airbursts, including ground/ice impacts, and their associated blast winds^33^ as major factors in the emplacement of Alaskan and Yukon mucks and their included megafaunal and botanical remains. In addition, we consider an astronomical scenario in which Late-Pleistocene episodes of terrestrial bombardment were caused by cyclic intersections with cometary debris during formation of the Taurid Complex^34^.

## “Muck” deposits

“Muck” is a miner’s term used to indicate frozen, usually dark-coloured, fine-grained sediments (silt), often containing large amounts of plant material and abundant vertebrate fossils that unconformably overlie gold-bearing gravels in stream valleys of Alaska and the Yukon Territory^6–9,16^. Mucks of the Fairbanks and Klondike districts are situated within the discontinuous permafrost zone (DPZ, >50% frozen ground; Fig. 1)^35^ and because of their high organic content^8,36^ emit a fetid stench upon thawing^6,9,37^. The silt portion of the mucks is generally inferred to be retransported (gravitationally emplaced) loess from nearby upland areas that was initially swept up by winds from glacial outwash plains and deposited across the landscape, primarily during periods of glacial expansion^8,38^. These loess beds have total thicknesses ranging from a few cm on ridges to >60 m at lower elevations^8,39^ and are generally referred to as “primary”, “upland”, or “air-fall” loess^40,41^ as opposed to the “muck”, “organic silts”, or “valley” and “retransported” loess of the valley bottoms^9,42^. Nearby volcanic centers of the Aleutians and Alaskan margin produced distal tephra beds intercalated within the mucks and primary silts, and these tephras have played an important role in determining the age and stratigraphic framework of these deposits^43^.

## Vertebrate and plant remains

**Figure 2:**
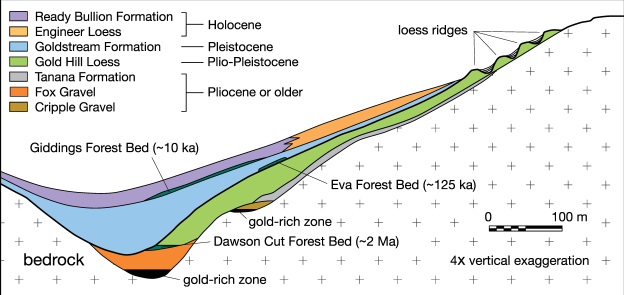
Schematic cross section of creek valley sediments near Fairbanks, Alaska^22^ showing stratigraphic relationships between primary loess (Gold Hill and Engineer Loess), retransported loess or “muck” (Goldstream and Ready Bullion Formations), forest beds (Dawson Cut, Eva, and Giddings), mass-wasting deposits (Tanana Formation), and gold-bearing gravel (Fox and Cripple Gravels; see Supplementary Information, “Quaternary stratigraphy”). Reproduced with permission of the Geological Society of America.

The fossil skull fragments assembled for this study are Wisconsinan in age (Table S1), so we will focus on information concerning emplacement of the corresponding Goldstream Formation (Fig. 2; see Supplementary Information, “Quaternary stratigraphy”), the equivalent King Solomon Formation (Silt unit) of the Klondike district, and their incorporated vertebrate and plant remains. Similar organic-rich loess deposits also occur in Siberia (Fig. 1) and are called “Yedoma”. Overall, the fossil bones recovered from these units show a spectrum of preservation ranging from highly weathered to exceptionally well preserved with intact ancient DNA^1,7,10^. The recovered mummified or “freeze-dried” partial carcasses also indicate that some part of the permafrost in the Fairbanks and Klondike areas has existed there since Wisconsinan time^6–9^. Moreover, soil horizons were often buried and frozen before the plant material could decompose, in some instances preserving it for >700 kyr^9,35^.

**Figure 3:**
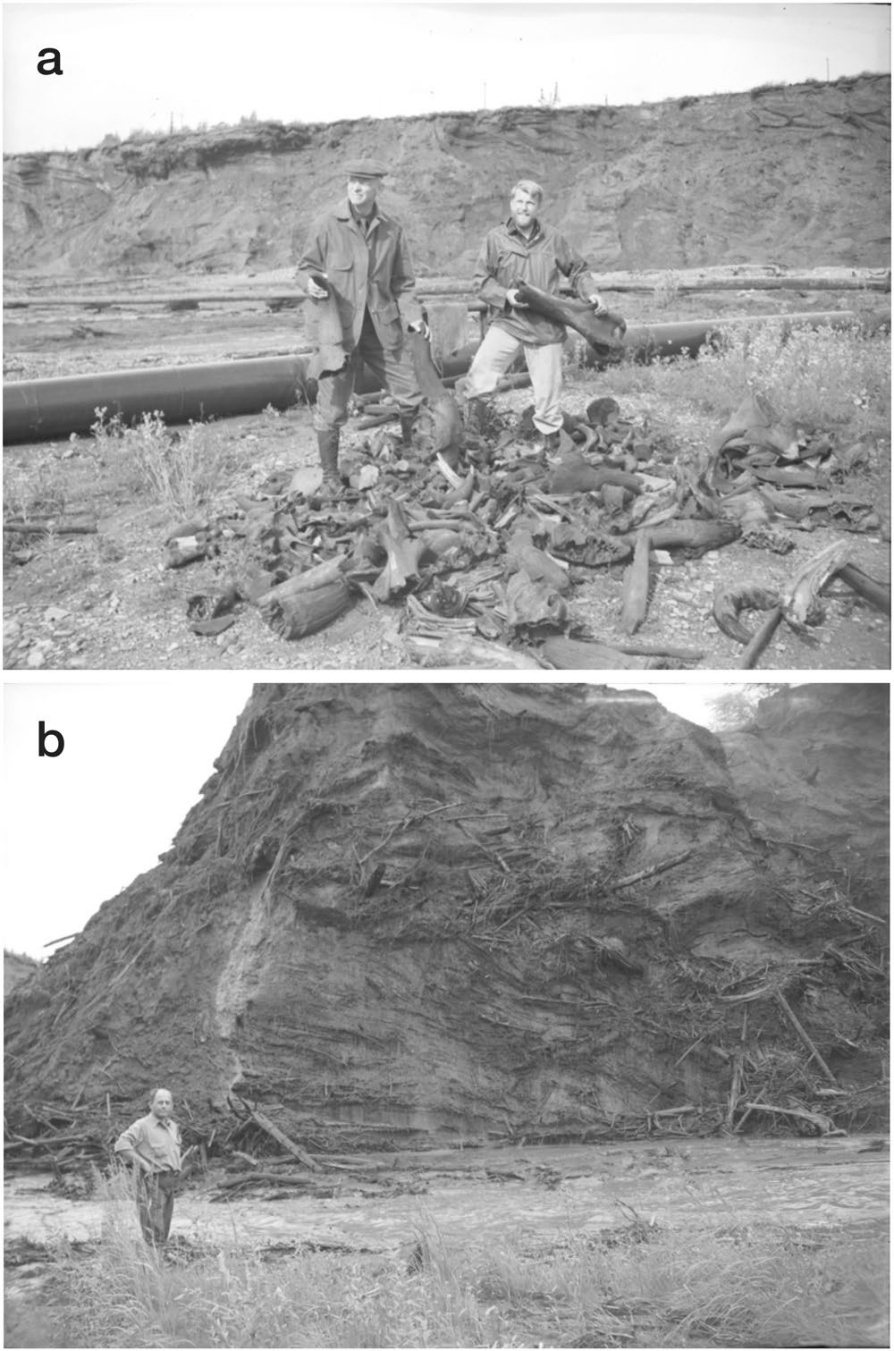
Photographs taken in 1941 at the Cripple Creek mine located just west of Fairbanks, Alaska. (**a**) Frank C. Hibben on August 3, at right, holding a broken mammoth’s humerus (*Mammuthus primigenius*) and an unidentified person holding bison skull fragments (*Bison priscus*) at one of Otto Geist’s fossil discard piles (see Supplementary Information, “Historical note”). Maxwell catalog no.: Hibben_Ak41_neg15bw. (**b**) Otto Geist on August 4 standing next to a runoff stream from hydraulic jets (monitors) used to thaw and wash away the muck overburden (background), and expose the gold-bearing gravels (see Fig. 2). Note the logs, branches, and other plant material protruding from the fined-grained frozen mucks. Maxwell catalog no.: Hibben_Ak41_neg16bw. Both photographs are from the Frank C. Hibben Photograph Collection, courtesy of the Maxwell Museum of Anthropology, University of New Mexico.

The remarkable preservation of vertebrate and plant remains within the mucks, however, is in stark contrast to the physical disruption and damage affecting much of this material. Much of the skeletal remains from Alaska and Yukon were disarticulated and broken prior to freezing (Fig. 3a), and the rare preserved carcasses were often mangled and torn apart^5,7,8^. Such dismemberment has been attributed to predators and scavengers, but these explanations raise questions including why the carcasses’ remaining fat and flesh had not been consumed^5^. In addition, the fine-grained character of the mucks differs markedly from that of the more massive logs, megafaunal bones and tusks enclosed within it. Although the valley-bottom mucks are thought to have accumulated from gradual downslope movement of primary upland loess by slopewash, creep, and mudflow^8,42^, this scenario appears inconsistent with the required rapid burial and freezing of well-preserved carcasses^7,8^ and plant remains^9,35^. Below we examine these and other paradoxes more closely, some of which amazed and bewildered early explorers, scientists, and miners venturing to the region.

### Fossil bone abundance

“Fossil bones are astonishingly abundant in the frozen ground of Alaska”^7^, and most observers from Quackenbush^14^ to Péwé *et al*.^22^ have made similar remarks. The majority of Alaskan fossils have been found in creek valley bottoms^8^, but the fossils have not been abraded or waterworn^7^. Bones are generally found throughout a given valley, and have been recovered from almost all creek valleys that have been mined^7^ (Fig. 3a). According to Otto Geist, most of the Pleistocene bones from the Fairbanks district were found in black muck containing plant remains^16^.

In the Klondike district, bones are mostly found near the base of the King Solomon Formation, usually within a few decimeters of its contact with the underlying auriferous gravel beds^9^. At one Alaskan locality, Little Eldorado Creek, 48 bison skulls and other related skeletal remains were found within a 35–m^2^ area^32^, while at other localities bones from a mixture of species have been recovered^7,8,18^. Although fossil remains are more abundant at some localities than others, many individual bones, or skeletal and carcass fragments, have been found in complete isolation^8,44^.

### Fossil bone dispersal and breakage

**Figure 4:**
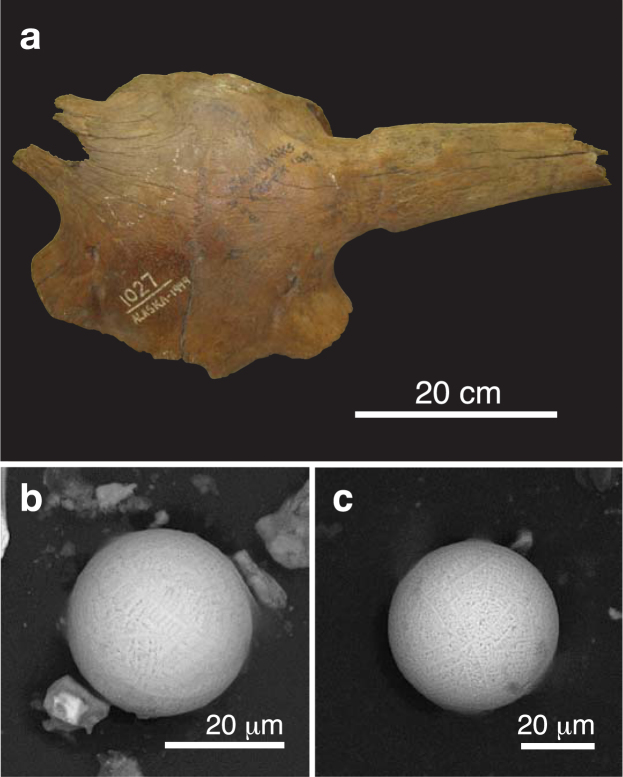
(**a**) Photograph of Alaskan bison skull A-501-1027 (*Bison priscus*) from the AMNH collection recovered at Fairbanks Creek in 1949. The skull has been dated at 18,635 ± 105 CAL yr B.P. (Table S1). (**b**,**c**) SEM images of two microspherules magnetically separated from fine-grained sediment contained within the skull.

**Figure 5:**
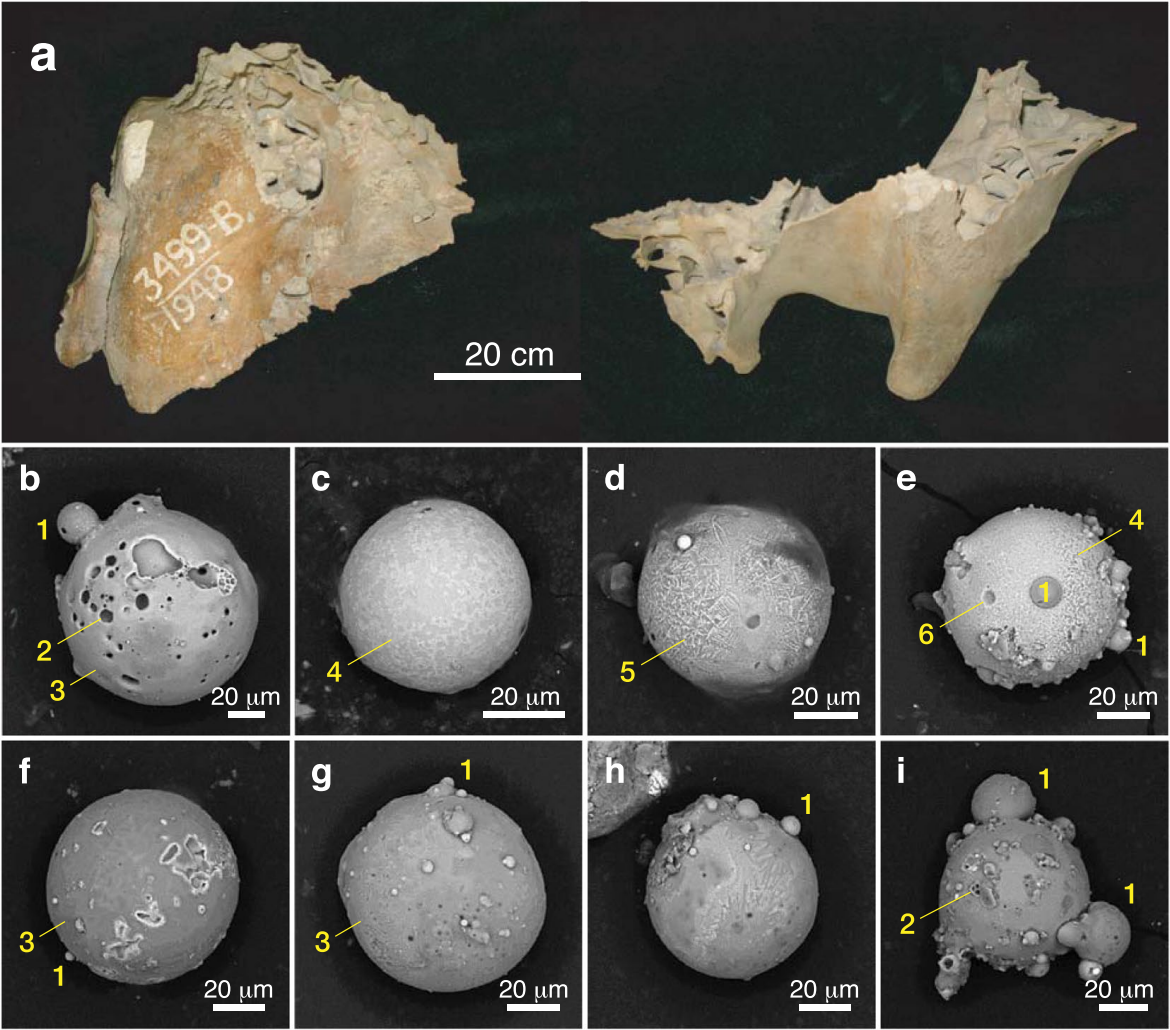
(**a**) Photograph of Alaskan mammoth skull fragments (A-478-3499; *Mammuthus primigenius*) at the AMNH collected in the Fairbanks mining district in 1948. The skull has been dated at 48,200 ± 2750 CAL yr B.P. (Table S1). (**b**–**i**) SEM images of microspherules recovered from fine-grained “muck” sediment encased within the skull fragments. Yellow numbers indicate (1) accretionary spherules, (2) vesicles, (3) smooth microstructures, (4) textured microstructure, (5) dendritic microstructure, and (6) possible microcrater.

**Figure 6:**
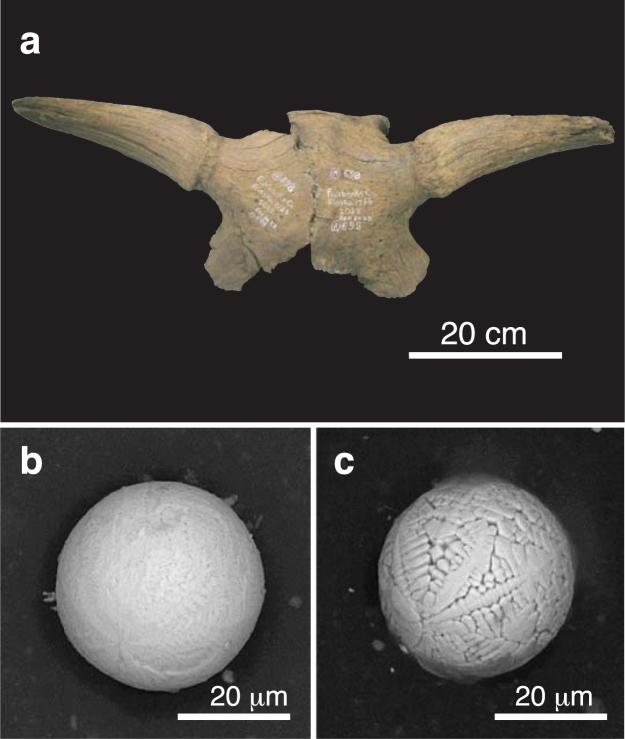
(**a**) Photograph of Alaskan bison skull (A-698-2022/2023; *Bison priscus*) from the AMNH collection recovered at Fairbanks Creek in 1954. The skull is undated. (**b**,**c**) SEM images of two microspherules magnetically separated from fine-grained sediment contained within the skull.

**Figure 7:**
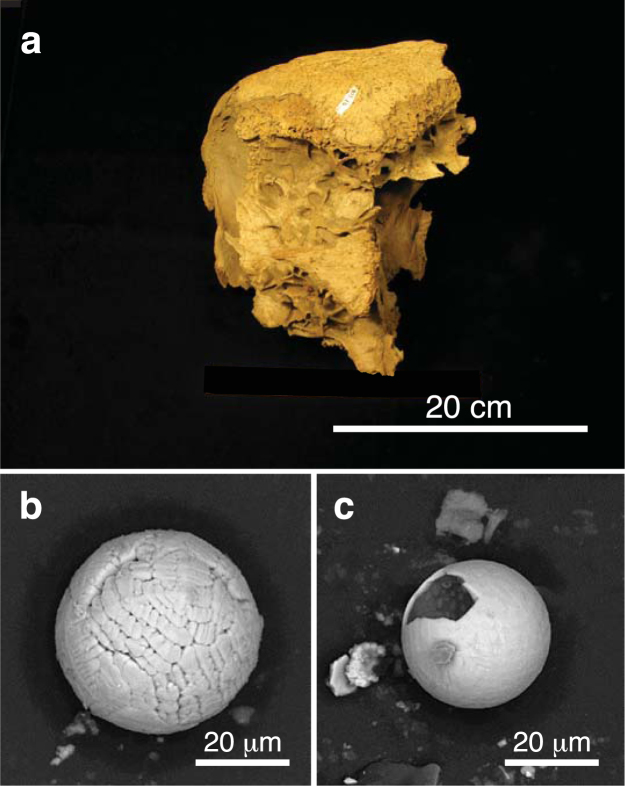
(**a**) Photograph of Yukon bison skull (Y-301.16; *Bison priscus*) from the Territorial Government collection in Whitehorse recovered at Homestake Gulch in the Klondike district. The skull has been dated at 29,065 ± 105 CAL yr B.P. (Table S1). (**b**,**c**) SEM images of two microspherules magnetically separated from fine-grained sediment contained within the skull.

“The dispersal of the bones is as striking as their abundance”^7^ and articulated bones are scarce in Beringia with the exception of small rodent carcasses (e.g., ground squirrels, pikas, mice) found underground within their nests and burrows^5,8^. Moreover, not only are individual megafaunal bones found separately, or mixed together in concentrated “bone beds”, they also are often broken (Figs 3a and 4–7, S1–S4). In examining large collections of Pleistocene fossil bones at the AMNH in New York and a Yukon Government warehouse in Whitehorse, we noted that the majority had been broken, including the largest (femora and humeri), with some still containing relict marrow, supporting the lack of predation. Because the mucks have apparently remained frozen since Wisconsinian time, dispersal and breakage of bones must have occurred prior to freezing and was not a general byproduct of the mining operations.

MacPhee *et al*.^44^ attempted to explain the dismemberment of Pleistocene carcasses and the resultant dispersal and breakage of bones recovered from Yedoma deposits on Russia’s Taimyr Peninsula as being caused by movements of ice in valley bottoms during spring breakup. They suggested that exposed limbs or other body parts of partially buried carcasses could have been sheared off by moving ice, and that ice-caused push fractures could have produced forces great enough to break bones as large as mammoth femora and humeri (see Fig. 3a). They acknowledged, however, that the “exquisite condition” of carcasses and many bones argues against these remains having spent a significant amount of time at the surface or having undergone any manner of “repeated redeposition”^44^.

### Muck sedimentology

A distinctive characteristic of the Beringian mucks is their uniform fine-grain size with only a few thin layers and lenses of coarser sand and gravel near their lower contacts^16^. Most mineral particles of the mucks will pass through a 200-mesh (<74 μm) sieve^6^, and, as mentioned above, the ultimate origin of these deposits is considered aeolian^7,9,22^. The Wisconsinian Goldstream mucks are poorly- to well-stratified, and the layers are conformable to the valley slopes and bottoms^16,42^. In the Silt unit of the Klondike district, planar bedding is the most common structure, as at Quartz Creek where a 17 m-thick section contains individual beds up to 5 cm thick. The silt can also be massive (≤3 m thick) with no apparent sedimentary structures^9^.

The sedimentary contrast of dispersed bone clasts (e.g., megafuanal skulls, tusks), isolated rock fragments ~2–10 cm in diameter^16^, and logs (Fig. 3b), within a silty matrix, however, is incompatible with a fluvial origin. A complete mammoth skull including tusks can weigh 100–150 kg or more, and a large male woolly mammoth tusk can weigh up to 80 kg^44^. Large volumes of water moving at high velocity would be needed to move such objects any distance, for which there is no water-related sedimentological evidence, indicating that the partial frozen carcasses have not been transported any distance by fluvial processes. Dense vegetal cover and low rainfall levels in Alaska have kept slopewash to a minimum^7^, and downslope movements of skeletal remains by creep and solifluction would be limited to the depth of seasonal thawing^6,7^, which is presently <1–2 m in the Fairbanks area^5^.

### Partial frozen carcasses

One of the most remarkable and perplexing aspects of the mucks is their preservation of frozen animal carcasses or mummies. The vast majority of megafaunal mummies in Beringia were found as partial carcasses, and the most commonly found body parts in Alaska have been isolated limbs of horses, bison, moose, and caribou^5^. Even the most complete mummies show signs of significant internal damage: the Siberian Berezovka mammoth had a number of broken bones (e.g., pelvis, ribs, foreleg, shoulder blade) and evidence of significant internal bleeding^5,45^. Notably, the well-preserved tip of a Steppe Bison’s tail (~22 cm long) was found under the sole of the mammoth’s right forefoot^45^. The headless Selerikan pony (Siberian) also had broken humeri and ribs, and the Steppe bison “Blue Babe” (Alaskan) had missing and broken bones including a shattered right tibia and broken right mandible^5^.

The nearly undamaged baby mammoth “Lyuba” from Siberia, found in 2007, also had a broken mandible^46^, whereas the baby mammoth “Dima”, found in 1977, showed a small wound on its right wrist and hemorrhaging of its heart muscle^5^. In both Dima and Lyuba, fine-grained mineral particles were found within their trachea, bronchi, and/or lung alveoli^5,46^. Only hide from the head, neck, and trunk, along with the attached left front leg of an Alaskan baby mammoth, “Effie”, were found at Fairbanks Creek in 1948^5,8^. Previously, in 1941, a leg from a different mammoth with bone, hide, and hair had been found near the Effie discovery site^16^. Full gastrointestinal tracts (e.g., Selerikan pony) and food material between their teeth (e.g., Berezovka mammoth) indicate that death for many of these mummies was “almost instantaneous”^5^. Furthermore, from their thick fat deposits and recovered gut contents, it appears that many of the animals died at roughly the same times of year during the late summer or autumn seasons^5^. Notably, megafaunal deaths were not concentrated during the lean months of winter when they would have been subject to harsher environmental conditions^8^.

### Plant material

The Goldstream Formation contains abundant layers, lenses, and clumps of plant material derived from grasses, mosses, alder, spruce, willow, cottonwood, and birch that match the present-day flora^6,8^. Pollen analyses indicate that trees during the Wisconsinan glaciation were scarce in the region and that the tree line was ~600 m lower, although forested areas likely prevailed at lower elevations within the creek bottoms^8^. Peaty layers are common in the Goldstream muck, ranging from <0.01 to >3 m in thickness^7,8^, and in many places contain tree stumps rooted in place with the trunks and treetops having been “broken off and carried away”^7,16^. Multiple forest layers (e.g., 6 horizons within a 6-m section^6^) are more common within the overlying Holocene Ready Bullion Formation^8^ (Fig. 2).

Severely splintered tree stumps and logs are often found in the Goldstream mucks, and the splintering has been attributed to freezing^7^, although many frozen stumps and logs, mostly lacking their bark, are well preserved^8^. Other buried wood includes sticks and branches ~2–13 cm in diameter (Fig. 3b)^8,16^. Some of the finely comminuted plant material found throughout most of the Goldstream muck is carbonized, with the total carbon content of this material being 2.39%^16^. Comminution of this plant material was thought to have resulted from a combination of forest fires, frost action, and downslope transport^16^.

## Results

The accelerator mass spectrometer (AMS) radiocarbon dates for the Beringian megafaunal skull fragments range between ~48 and 18 ka B.P. (Table S1) and are similar to those commonly determined for megafaunal bones and carcasses recovered from Alaskan and Yukon mucks^20,25,47^ and Siberian Yedoma deposits^5,25,47^. The youngest age (18,635 ± 65 yr B.P.) is for an AMNH bison skull with a shellacked exterior (Fig. 4a). The shellacked top of the core drilled from this skull was removed before processing, but contamination of the remaining bone sample remains a possibility. The mammoth skull had also been shellacked, but its dating sample was collected from bone structures within the skull (Fig. 5a).

Magnetic fractions extracted from aliquots of bulk sediment removed from each skull ranged in size from ~5–44 g/kg, averaging 23.6 g/kg (Table S1). Upon examining each fraction for microspherules, two fractions were found to contain only rounded detrital magnetite grains (Y-404.716, Y-109-8; Fig. S4), whereas the other seven contained numerous microspherules ranging in abundance from ~1000–18,000/kg, with an average of ~8000/kg. For comparison, concentrations of magnetic spherules (~10 μm–5.5 mm in diameter) from samples of the Younger Dryas boundary layer collected at 18 sites in the northern hemisphere range from 5–4900 spherules**∕**kg with an average of 940*/*kg^30^. Only one rounded quasi-spherical particle was found within the magnetic separate from primary loess sample AK-915. It was lost before it could be identified, but if similar to the other recovered microspherules it was present at a much lower abundance (~17/kg) by several orders of magnitude (Table S1).

Scanning electron microscopy (SEM) images (see Supplementary Information, “Analytical methods”) for some of the 49 muck microspherules selected in our study are shown in Figs 4–7, S1–S3. Their shapes and surface textures are similar to those of microspherules associated with the Chixulub impact at ~66 Ma^48^, the Chesapeake Bay impact at ~35 Ma^49^, the Meteor Crater impact at ~50 ka B.P., the Younger Dryas boundary layer at ~13 ka B.P., the Tunguska airburst in 1908, and the Trinity atomic airburst in 1945^30,50^. In particular, microspherules from the mammoth skull (Fig. 5b,e,g–i) show accretionary features, which form within the impact plume when partially molten and still tacky projectiles collide at low differential velocities. At higher initial velocities destructive collisions often occur resulting in particles displaying microcraters (Fig. 5e) and/or brittle fracturing^30^.

**Figure 8:**
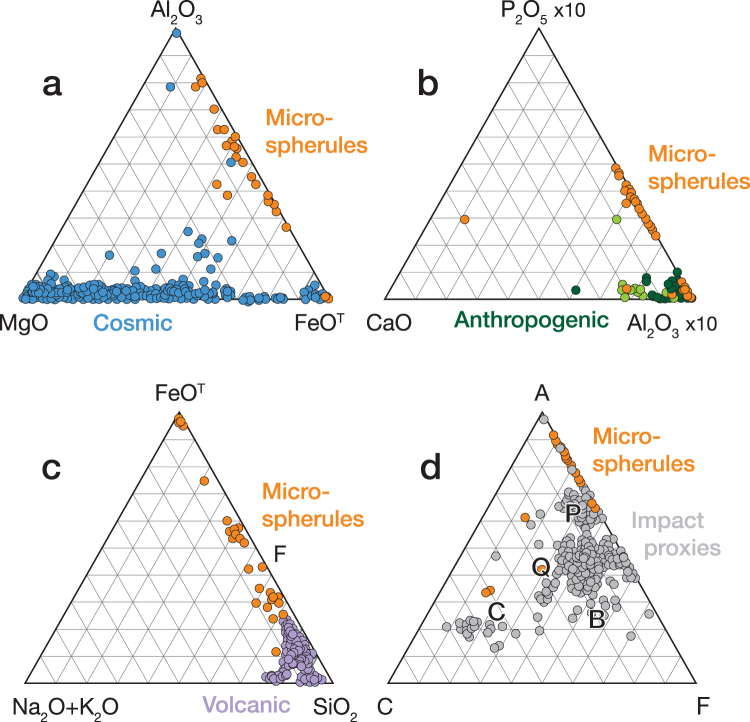
Ternary diagrams (molar values of oxides) comparing 49 representative Beringian microspherules (orange dots) to (**a**) cosmic material (blue dots; *N* > 700); (**b**) anthropogenic fly ash (light green dots; *N* = 225) and other anthropogenic spherules (dark green dots; *N* = 42); and (**c**) volcanic materials (purple dots; *N* > 10,000). (**d**) Standard ACF (Al_2_O_3_-CaO-FeO) ternary diagram comparing the microspherules to cosmic impact materials, including spherules and tektites from twelve accepted impact craters and strewnfields, including the Australasian tektite field (~780 ka) and the Cretaceous-Paleogene impact layer (~66 Ma) (*N* > 1000). The microspherules are heterogeneous as indicated by the letters representing specific metamorphic rock types: P, pelitic, e.g., clayey mudstones and shales; Q, quartzo-feldspathic, e.g., gneiss and schist; B, basic, e.g., amphibolite; and C, calcareous, e.g., calcite (*N* = 43). Formulas for diagram: A = (Al_2_O_3_ + Fe_2_O_3_) − (Na_2_O + K_2_O); C = (CaO − (3.33 × P_2_O_5_)); F = (FeO + MgO + MnO).

Energy dispersive X-ray spectroscopy (EDS) analyses (see Supplementary Information, “Analytical methods”) of the 49 microspherules indicate two distinct chemical populations: those from the mammoth skull (Fig. 5) are predominately aluminosilicate (Al_2_O_3_ = 30.7 wt.%, SiO_2_ = 34.4 wt.%, FeO = 23.4 wt.%, CaO = 2.9 wt.%, with all other oxides being <2.3 wt.%), whereas those from the remaining six bison skulls (Figs 4, 6–7, S1–S3) are typically iron-rich (FeO = 87.4 wt.%, Al_2_O_3_ = 2.0 wt.%, SiO_2_ = 2.3 wt.%, CaO = 4.0 wt.%, with all other oxides being <1.1 wt.%). Various oxides of the 49 microspherules are plotted on ternary diagrams along with those for known populations of other types of spherules. In Fig. 8a, abundances of Mg, total Fe, and Al for the 49 microspherules are compared with >700 cosmic spherules (melted micrometeorites) and micrometeorite particles from 83 sites, mostly in Antarctica and Greenland. The cosmic material tends to be significantly more enriched in MgO and depleted in Al_2_O_3_ relative to the muck microspherules. In Fig. 8b, compositions of the 49 microspherules are compared to 267 fly-ash particles and anthropogenic spherules collected at 48 sites around the world that were produced primarily by coal-fired power plants^30^. In this case, the fly ash is enriched in Al_2_O_3_ and far more depleted in P_2_O_5_ relative to the muck microspherules. The potential for anthropogenic contamination, however, is extremely low because it is usually restricted to near-surface deposits of industrial age (<300 yr), whereas the Beringian fossils had remained deeply buried and frozen for thousands of years.

A comparison of the muck microspherules with >10,000 volcanic samples (glass, tephra, and spherules) from 205 sites of global extent is shown in Fig. 8c. Volcanic material tends to be enriched in silica (>40 wt.%) and depleted in Fe abundances compared to the 49 microspherules. Moreover, the sediment removed from the skull fragments lacked any typical indicators of volcanic activity such as volcanic ash and/or tephra. Finally, compositions of >1000 impact-related markers (spherules, ejecta, and tektites) from 12 craters and tektite-strewn fields^30^ were compared to compositions of the muck microspherules (Fig. 8d). In addition, compositions of typical metamorphic rocks (P, pelitic; Q, quartzofeldspathic; C, calcareous; and B, basic) are indicated on the ternary diagram. The 49 microspherules are most similar to pelitic rocks that are chemically equivalent to clayey mudstones and shales with several having more calcareous compositions. Biasing of the microspherules toward more Fe-rich compositions (A and F), however, was caused by their magnetic separation from the bulk sediments. In summary, approximately 75–90% of the muck microspherules are dissimilar to known cosmic, anthropogenic, and volcanic spherules and related materials, and are most similar to known impact-related spherules and classes of terrestrial sediments.

Average crustal abundances are 0.51 ppb for platinum (Pt) and 0.52 ppb for palladium (Pd), based on sediment and loess data^51^, and average values for our primary loess samples are 1.02 ppb for Pt and 1.67 ppb for Pd, with a lower detection limit of 0.1 ppb (Table S2). For muck samples from three of the four bison skulls analysed, Pt abundances are elevated and range from ~2–4 ppb; for skull Y-404-716, in which no microspherules were found (Table S1), the sediment’s Pt content (0.90 ppb) is similar to that of the primary loess (1.02 ppb). Pd values remain relatively constant between the muck and loess samples, averaging 1.31 ppb, and the Pt/Pd ratios are thus higher for the Pt-rich muck samples. High Pt/Pd ratios suggest an influx of impact-related, Pt-enriched material from an impactor and/or from Pt-rich target rocks, possibly included within the microspherules. As an example, Andronikov *et al*.^52^ found high Pt abundances (18–460 ppb) in four of six microspherules collected from the Younger Dryas Boundary layer at Blackwater Draw, NM.

## Discussion

The high-temperature, melt-quenched and accretionary microspherules, and Pt enrichments associated with sediments from the megafaunal fossils of this study (Figs 4–7, S1–S3), are well-established indicators of extraterrestrial impact events^30,50,53^. In light of this evidence, much of the death, dismemberment, burial, and preservation of megafaunal bones and partial carcasses within the frozen Beringian mucks is perhaps better explained by the catastrophic effects of cosmic impacts rather than by more commonplace causes of mortality such as illness, accident, advanced age and/or predation. In the following discussion, we reinterpret parts of the mucks and their contents as blast deposits and attempt to resolve the apparent paradoxes and inconsistencies outlined above. Examples to be applied come from studies of blast effects associated with the ~50 ka B.P. Meteor Crater impact^33^, 1908 Tunguska airburst^54^, ≥1945 nuclear tests^55^, and 1980 Mount St. Helens eruption^56^.

The blast or shock waves associated with ground impacts or airbursts cause an almost instantaneous rise in air pressure to peak overpressures as the wave fronts expand at supersonic velocities. The subsequent mass movement of air generates blast winds, which travel more slowly than the blast waves but can be just as damaging, if not more so^33,57^. In general terms, the area (*A*) damaged by an explosion is proportional to the cube root of its yield (*E*) squared^33^, or *A* ∝ *E* ^2/3^. The amount of damage is also a function of the explosion’s height, and for a given yield there is an optimum blast height at which the damage is maximized. For the 1908 Tunguska airburst, the blast height was ~6–9 km^58^, near the optimum height for a ~10–20 megaton (MT) blast, and trees of the Siberian taiga were knocked down^33^ over an area of ~2150 km^2^. Intense thermal radiation was also associated with the Tunguska event. Within a radius of ~10–15 km from the epicenter the sides of trees facing the atmospheric impact were scorched, and ignited treetops were reported to have burned in many places for up to 24 hr^54^.

Kring^33^ quantified the air blast for the Meteor Crater impact using scaling relationships derived from nuclear bomb tests. Given an estimated explosive yield of ~20–40 MT for the impact of an ~50-m-diameter iron asteroid^59^, the air blast would have flattened trees within a ~16–22 km radius of ground zero, and damaged them over an area of ~4100–8500 km^2^. Within a radius of ~3–5 km, the ephemeral blast wind (>1000 km/hr) would likely have “scoured the surface of loose debris, plants, animals, and soil”, and, although decreasing in intensity with distance, would have remained “fairly large” up to distances of ~20–40 km^33^. Both types of impact events would have killed megafauna from various levels of blast injuries.

Primary blast injuries are caused by the nearly instantaneous change in environmental pressure from passage of the blast wave. In large mammals, the organs most commonly affected are the lungs, ears, bowel, and central nervous and cardiovascular systems^57^. The blast wave can also fracture bones and cause dismemberment including traumatic amputations and decapitations. From the exposure of a wide range of animals to nuclear tests (e.g., steers, burros, sheep, goats, monkeys, pigs, dogs, cats, rabbits, chickens, rats, hamsters, and mice), it was determined that larger animals generally endure higher overpressures better than smaller ones, and that animals affected by primary blast injuries usually die within a few minutes^55^.

Secondary blast injuries are associated with the subsequent blast wind and result from projectiles that have been picked up and energized by it. Sufficiently large fragments can also cause direct limb amputations and decapitations. Tertiary blast injuries result when victims are hurled against the ground or fixed objects. Often, victims tumble along the ground sustaining multiple injuries. Miscellaneous blast injuries include inhalation of dust, and burns from the explosion itself or from fires ignited by it^57^.

A modern example of a catastrophic explosion causing death and dismemberment of large mammals was the 1980 eruption of Mount St. Helens. An equivalent explosive yield for the eruption is estimated to have been ~35 MT, and the “blast zone” of downed trees and destroyed habitat^56^ covered an area >350 km^2^. Animals within the blast zone died from suffocation, severe blast injuries, and falling timber and/or pumice blocks^60^. Of the 24 sites where remains of elk (*Cervus elaphys*) and black-tailed deer (*Odocoileus hemionus*) were found, 5 were examined ~1 year later by Lyman^56^. All 5 sites were located ~15 km away from the Mount St. Helens vent, and at this distance mature trees had been snapped off and splintered near their base and completely stripped of their branches. The volcanic crater could not be seen from two of the sites, where intervening ridges probably acted as deflectors, but the crater was visible at the other three sites where they would have been subjected to the full force of the blast^56^.

Portions of cervid skeletons were found buried under ~20–60 cm of volcanic ash and no soft tissue was preserved. Long bones at the sheltered sites were not broken, but those at the exposed sites were fractured, in some cases shattered, and were generally more disarticulated and widely scattered. Bone fracturing was nonselective as unbroken examples of all skeletal elements could be found. Apparently the elk and deer at the exposed sites were engulfed in the eruption’s ash cloud, hurled along with trees, rock fragments and pumice blocks, and deposited some distance away at their recovery locations. Carnivore damage to the bones was minimal because many of the bones were buried, and scavengers within the blast zone would have been killed as well^56^.

The blast injuries sustained by cervids at Mount St. Helens apparently match much of the damage exhibited by the vertebrate remains found within Beringian muck and Yedoma deposits. Broken and shattered bones, especially within the frozen mummies (e.g., Berezovka mammoth, Lyuba, Blue Babe), have been attributed to retransportation and diagenetic processes^5,46^. Pfizenmayer^45^, however, noted that bleeding had occurred at the break of the Berezovka mammoth’s right foreleg, and inferred it had still been alive at the time. The mammoth, therefore, was initially thought to have fallen into a “crevasse”^45^, but instead could have been thrown and/or tumbled across the ground by blast winds, come to rest in a surface depression or pit, and been covered over by blast-mobilized sediment. The segment of a bison’s tail found under the Berezovka mammoth’s foot could simply have been clipped off a nearby grazer by the blast and deposited along with the mammoth’s carcass. That a healthy bull mammoth was instantly killed while grazing, subjected to multiple broken bones and internal injuries, and rapidly buried along with a body part from another animal, is consistent with the effects of a large explosive event.

The smallest frozen mummies preserved in underground nests and burrows (e.g., ground squirrels, pikas, mice) could have been killed because of their higher susceptibility to blast overpressures. At the surface, baby mammoths (e.g., Dima, Lyuba), with smaller cross sections, would have sustained less blast damage than adults. The baby mammoth Effie, however, could have been blown apart in closer proximity to an explosive center. The disarticulated and scattered bones along with partial carcasses and the relatively abundant mummified limbs are likely indicative of the damaging effects of airbursts and/or ground impacts. The overall abundance of Beringian fossil bones, often including those of different species within a single bed, do not conform with normal modes and rates of attrition, but are in accord with the expected aftermaths of cosmic impact events.

The mixture of vertebrate remains, plant debris, and loess within the Beringian mucks is consistent with ground-surface scouring, transport, and the chaotic redeposition of these materials in creek valleys by blast winds (Fig. 3b). Trees exposed to such winds would have been stripped of their branches, leaves, and bark, and either knocked down or broken off and splintered, like those found in the muck deposits. The finely comminuted plant material distributed throughout the mucks might have resulted from pulverization of less durable plant debris (leaves, grasses, bark, etc.) within turbulent blast winds, and its carbonization could have been initially caused by scorching and/or burning from intense thermal radiation associated with atmospheric passage of hypervelocity impactors. Stacked layers of killed forests indicate that creek-valley silt accumulation was not gradual or uniform, but that it was deposited cyclically in large amounts over short intervals of time. In addition, less severely injured animals could have suffocated in the dense clouds of airborne silt (e.g., Dima, Lyuba).

**Figure 9:**
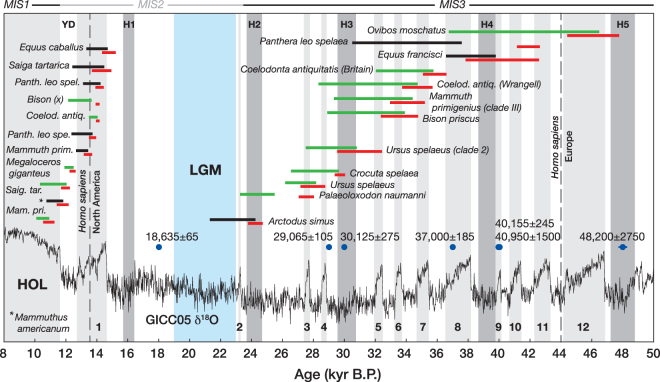
Megafaunal regional or global extinction-event age ranges, as identified by Cooper *et al*.^25^ in Late Pleistocene Eurasian and North American ancient DNA and paleontological data sets, plotted along with climate intervals determined from the GICC05 δ^18^O ice-core record (black curve) from Greenland^71,72^. Red bars, with taxonomic names, indicate the youngest AMS ^14^C dates for the events (±2 SD), and green (Eurasia) and black (North America) bars indicate GRIWM-based estimates of last-occurrence temporal ranges (95% confidence intervals); the Gaussian-resampled, inverse-weighted McInerney (GRIWM) method incorporates sampling density and dating errors in order to estimate the most plausible temporal ranges^25^. Light gray boxes (1–12) indicate Dansgaard-Oeschger (D-O) interstadial warming events^73^, and darker gray boxes indicate Heinrich cold events (H1-H5)^74^. Blue dots and values represent the AMS ^14^C dates for the skull fragments of this study (Table S1), the light blue area delimits the Last Glacial Maximum (LGM), HOL abbreviates the Holocene Epoch, YD denotes the Younger Dryas stadial, and ranges for Marine Isotope Stages (MIS) 1–3 are shown atop the plot. Reproduced with permission of the American Association for the Advancement of Science.

The buried forest layers, range of radiocarbon dates, and microspherules associated with the vertebrate fossils indicate that deposition of the Alaskan and Yukon mucks would have required more than one impact event between ~48 and 18 ka B.P. (Fig. 9; Table S1). A possible mechanism for repetitive impacts on Earth is the cyclical intersection of its orbit with meteoroid streams containing numerous objects large enough (~10–100 m) to generate Tunguska-class airbursts and ground impacts. Since the late 1970s, a group of British astronomers^34,61,62^ has calculated that such a scenario likely affected Earth throughout Late Pleistocene time during formation of the Taurid Complex.

The massive Taurid Complex is known to consist of several dynamically related meteoroid streams, several Apollo-type asteroids, and Comet 2 P/Encke, which has a ~5-km diameter and orbital period of 3.3 yr^61,63^. These objects are most likely the remnant debris of a large comet (~50–100 km) from the trans-Neptunian region that was injected into a short-period, sub-Jovian, low-inclination Earth-crossing orbit^63,64^ by gravitational fields of the outer planets. Such injections of large comets into the inner solar system possibly occur at a mean rate of roughly once per 30 to 100 kyr^64^.

Spontaneous splitting is a major factor in comet disintegration and may occur anywhere along its orbit, with some preference for near-perihelion breakup, and would lead to hierarchical fragmentation of the progenitor comet into numerous ~1 km-sized bodies in short-period orbits, many smaller objects in the ~10–100 m range, and large quantities of dust^34,64^. Substreams of meteoroids having similar but separate orbits within the complex are evidence of this cascading mode of fragmentation, which would likely have occurred over a period of ~10–100 kyr^63,64^. Using the observed dispersion of material in the Taurid Complex, Steel and Asher^62^ have calculated an overall age of at least 20–30 kyr, similar to the older age limit of megafaunal fossils containing impact-related microspherules. The continued dispersal of Taurid material by collisions, and by gravitational and radiative effects, has created a broad sporadic stream surrounding the complex^65^.

Jupiter’s gravity would predominantly cause the Taurid-object orbits to precess, and dynamical calculations have shown that Earth would encounter these orbits at ~3000 yr intervals^34,64^. Over the ~30-kyr period spanned by the skull-fragment ages (Table S1), it is expected that one or more encounters might have individually generated the atmospheric impact of hundreds to thousands of Tunguska-sized objects on a hemispheric scale. Such bombardment episodes could have lasted for several hours and occurred at certain times of the year, every few years, over a period of one to two centuries^34,64,65^.

The Late Pleistocene megafaunal extinction events appear to have occurred over a time interval between ~46 and 24 ka B.P. prior to the LGM, and again over a shorter interval between ~15 and 11 ka B.P. near the end of the Pleistocene Epoch^47,66^. Recently, Cooper *et al*.^25^ investigated all megafaunal species, or major clades, with comprehensive radiocarbon-dated series, to determine the timings of regional or global extinctions, and invasions or replacements by conspecific or congeneric populations (Fig. 9). They determined 31 such events widely distributed across Eurasia and North America that involved animals having diverse ecological roles and life histories^25^. The lack of megafaunal extinction events during the LGM, and to a lesser extent the YD stadial, suggests that cold environmental conditions were not an important driver of the extinctions, and Cooper *et al*.^25^ found that the population transitions were instead significantly correlated with rapid climate shifts associated with the Dansgaard-Oeschger (D-O) interstadial warmings (Fig. 9). Because of the apparent lack of ecological turnovers related to similar interstadial events that frequently occurred before ~46 ka, when modern humans were mostly absent from Eurasia and North America, Cooper *et al*.^25^ concluded that humans likely played a significant role in enhancing the effects of rapid climate change on Late Pleistocene megafaunal extinctions and population transitions^25^.

The presence of two geochemically distinct microspherule populations found by us in Late Pleistocene skull fragments points to at least two episodes of cosmic impact, and/or different target source rocks, and the skull ages (~18–48 ka B.P.; Table S1; Fig. 9) indicate that perhaps a greater number of such episodes (~2–4) occurred. It is also unclear how many separate extinction events there were both before and after the LGM. Cooper *et al*.^25^ noted a distinct cluster of events between ~37 and 32 ka, corresponding to D-O interstadials 5–7, and the question arises whether these extinctions with overlapping ages could have resulted from a single short-lived event. By comparing our ^14^C dates with the extinction age ranges in Fig. 9, it appears plausible that there could have been fewer discrete impact-related extinction events prior to the LGM, possibly occurring around 40–42 ka, 34–37 ka, and 27–30 ka, with another at the onset of the YD stadial (~13 ka)^26–28^.

Modern humans have also been implicated in the Late Pleistocene megafaunal extinctions^23–25^, but the temporal correspondence between these events both in Eurasia and North America seems to argue against their involvement (Fig. 9). The megafaunal extinctions and population transitions during MIS3 occurred in North America before modern humans had arrived, and in Eurasia over an extended period well after their arrival, whereas the terminal Pleistocene extinctions (MIS1-2) appear roughly coincident with modern humans’ arrival in North America, but occurred in Europe long after they had become established there. It would appear that some mechanism other than intensive human hunting drove the extinctions in both locations over the same time intervals. Moreover, the lack of evidence for ecological disturbances associated with interstadial warmings prior to ~46 ka^25^ suggests that rapid climate change might also have played a more limited, if any, role in the Late Pleistocene megafaunal extinctions.

The range in degree of fossil preservation, from weathered bones to frozen mummies, likely indicates the depth and/or timing of burial. Mummified carcasses and body parts were probably rapidly buried and frozen at deeper levels, whereas well-preserved bones were buried close enough to the surface to allow decomposition of the attached soft tissues before freezing, while weathered bones were exposed at the surface before burial by subsequent depositional events. Fraser and Burns^9^, however, noted “the position of bones of various ages near the lower contact of the Silt unit suggests…redeposition by events of considerable magnitude”. With the lack of evidence in the mucks for fluvial transport, such redeposition might have been accomplished by one or more impact-generated blast winds. During a bombardment episode, blast winds could have excavated and mixed bones of various ages, including those from animals that had already died of more common causes. Thus, the extensive breakage and disarticulation of skeletal elements in the Alaskan and Yukon mucks could have resulted from a relatively few episodes of cosmic bombardment.

## Conclusions

Our results suggest that large amounts of melt-quenched impact spherules, associated with Pleistocene megafaunal remains, were deposited in the mucks of eastern Beringia after ~48 kyr. The SEM/EDS and Pt/Pd data we acquired indicates they are not cosmic, anthropogenic, or volcanic in origin (Fig. 8), but were most likely produced by hypervelocity airbursts and ground/ice impacts. Based on this evidence, and the damaged and disrupted character of the muck’s vertebrate and botanical material documented by others^6–9^, we conclude that some parts of the Beringian muck and Yedoma deposits were catastrophically emplaced during the Late Pleistocene by blast winds associated with multiple episodes of cosmic impact.

Blast winds from the impacts would have swept across the Beringian landscape flattening trees and killing, dismembering, and burying megafaunal carcasses or body parts, along with logs, branches, other plant material, with a matrix of redeposited loess in low-lying creek valleys. Normal depositional processes such as slopewash, creep, and mudflow would have continued within the valleys during the thousands or more years between blast events. The lack of microspherules in the primary loess (Table S1) indicates that it was emplaced between impact episodes and those microspherules found within the mucks were added to the remobilized primary loess as it was retransported by blast winds along with comminuted organic material and the damaged and fragmented vertebrate and plant remains.

The radiocarbon dates determined for the skull fragments of this study are too few to support any robust correlation with the Late Pleistocene extinction events^25^. Further studies of fossils with well-established stratigraphic contexts are needed before the actual role of impacts in driving or contributing to the megafaunal extinctions can be inferred. Elevated Pt abundances^53,67^ and microspherules^26,30,50,52^ have also been associated with the YD-onset impact event^26–28^, and our results suggest that the terminal megafaunal extinctions were the culmination at ~13 ka B.P. of a relatively small number of subsidiary episodes that occurred prior to the LGM during MIS3 (Fig. 9). Chaotically deposited arboreal plant material and the presence of megafaunal carcasses within the Ready Bullion and coeval upper Organic mucks of Alaska and the Yukon Territory (Fig. 2), respectively, suggest that extraterrestrial impacts might have continued into the Holocene Epoch.

## Methods

The skull fragments assembled for this study are from Pleistocene vertebrate fossil collections curated at the American Museum of Natural History (AMNH) in New York City (Figs 4–6) and at the Yukon Government offices in Whitehorse (Fig. 7, S1–S4). Although collection locations are known for most of the fossils, they lack any useful stratigraphic information. Although the Yukon fossils had been subjected to little or no sample preparation and cleaning, the surfaces of the AMNH fossils had been cleaned and shellacked. In both cases, however, the selected skulls still contained significant amounts of host sediment, or dried muck, within them, and the included sediment was collected to determine if it contained any markers of extraterrestrial impact^30,50^. The fine-grained sediment was shaken from each skull onto fresh pieces of 8^1/2^ × 11-inch (21.6 × 27.9 cm) white copier paper, and then transferred to new plastic sample bags. Bone samples for radiocarbon dating (see Supplementary Information, “Analytical methods”) were taken from seven of the nine skulls using either a small handsaw or electric drill and hole-saw. Optimum samples for dating from two of the skulls (Table S1) were not obtained because they lacked enough thick, dense, and unweathered bone.

D. Muhs (USGS) collected samples of primary loess (Gold-Hill type), of equivalent and younger age to the mucks (Table S1), at the Halfway House, Chena Hot Springs Road, and Birch Hill sections near Fairbanks, Alaska. The geochronology of the Chena Hot Springs Road and Birch Hill sections is based on correlations with dated tephra and radiocarbon dates on wood, charcoal, or organic matter from soil horizons^40,41^. Ages were assigned to the samples from these sections due to their close proximity to dated sediment horizons or from interpolation between them. Ages for samples from the Halfway House section were similarly assigned based on stratigraphic relations with dated tephra and geomagnetic-field-excursion horizons, and with thermoluminescence-age determinations^68,69^.

Magnetic separates were extracted from bulk sediment slurries using a large neodymium (NdFeB) magnet (5.15 × 2.5 × 1.3 cm) wrapped in 4-mil plastic bags^30,50^. Each magnetic grain fraction was dried, weighed, and sorted into four size sub-fractions of >150 μm, between 53 μm and 150 μm, between 38 μm and 53 μm, and <38 μm using a stack of ASTM sieves. Aliquots of each grain-size fraction were then examined under reflected light using a 200x–300x zoom microscope to manually count and photograph the candidate microspherules (Fig. S5). Microspherules are defined as being <2 mm in diameter, and those of this study were usually found within the smaller two size fractions of the Alaskan and Yukon muck samples. Forty-nine candidate microspherules from the mucks were mounted on SEM stubs for further analysis.

Details of the SEM and EDS techniques, AMS radiocarbon dating, and platinum and palladium analytical methods are given in the Supplementary Information, “Analytical methods”. The datasets generated and analysed during the current study are available from the corresponding author on reasonable request.

## Electronic supplementary material

Supplementary Information
