# Supplementary material for: Impact-related microspherules in Late Pleistocene Alaskan and Yukon “muck” deposits signify recurrent episodes of catastrophic emplacement
Source: Sci Rep. 2017 Nov 30;7:16620. doi: 10.1038/s41598-017-16958-2 (PMC5709379; doi:10.1038/s41598-017-16958-2)
Supplement: Supplementary file 1 — Supplementary Information [file 41598_2017_16958_MOESM1_ESM.pdf]

Supplementary Information:

Impact-related microspherules in Late Pleistocene Alaskan and Yukon "muck" deposits signify recurrent episodes of catastrophic emplacement

Jonathan T. Hagstrum[1,*], Richard B. Firestone[2], Allen West[3], James C. Weaver[4], and Ted E. Bunch[5]

[1]U.S. Geological Survey, Menlo Park, CA 94025, USA

[2]Lawrence Berkeley National Laboratory, Berkeley, CA 94720, USA

[3]Comet Research Group, Prescott, AZ 86301, USA

[4]Wyss Institute for Biologically Inspired Engineering, Harvard University, Cambridge, MA 02138, USA

[5]School of Earth Science and Environmental Sustainability, Northern Arizona University, Flagstaff, AZ 86011, USA

*To whom correspondence should be addressed. Email: jhag@usgs.gov

Quaternary stratigraphy

The Quaternary stratigraphy of surficial deposits in central Alaska is similar to that in the western Yukon Territory and is depicted schematically in Figure 2. The regional bedrock is mainly Paleozoic to Mesozoic schist, slate, and gneiss with local masses of basalt, quartz diorite, and granite[S1]. The oldest unconsolidated deposit is the gold-bearing Cripple Gravel of late Tertiary age[S2-S3], and the next youngest unit is the poorly stratified auriferous Fox Gravel that includes an occasional waterworn megafaunal bone or wood fragment[S4]. Equivalent to the Alaskan gravels are the Late Pliocene White Channel and Early Pleistocene Klondike Gravels of the Yukon Territory[S5]. The Tanana Formation is thought to be contemporaneous with the Fox Gravel, and is a ~1–25 m-thick inactive solifluction layer, which is comprised of schist and quartz fragments within a sandy matrix[S2,S6].

Overlying the Tanana Formation is the Gold Hill Loess (Fig. 2), composed of thick frozen layers of massive aeolian silt, emplaced between ~2 Ma and ~130 ka B.P., that makes up the majority of loess beds in the Fairbanks region[S2,S7]. Muhs *et al.*[S8-S9], however, have sampled upland loess sections in the Fairbanks region equivalent in age to the younger Goldstream Formation (see below), indicating that Gold Hill-type loess can be younger than ~130 ka. The Gold Hill Loess represents several intervals of deposition and erosion, and major unconformities divide the formation into three members. The upper two members include a number of tephra beds, which aid in identifying these units, but no tephras have been found in the lower member[S6-S7]. Remains of small arctic and alpine mammals are found throughout the Gold Hill Loess, and, although megafaunal bones are included, no frozen carcasses have been found within it[S6]. At the base of the

Gold Hill Loess's middle member is the Dawson Cut Forest Bed (~2 Ma), which is a ~1–3 m thick layer containing peat lenses, logs and forest beds[S7].

The Goldstream Formation (Fig. 2), also known as the Goldstream "muck"[S4], unconformably overlies the Gold Hill Loess and locally, on lower slopes, the Eva Forest Bed[S10], Fox Gravel, or Tanana Formation[S2]. One of the most widespread formations in valley bottoms of central Alaska, the Goldstream Formation has been called the "most interesting" by Péwé[S2] and is also perhaps the most enigmatic. The formation consists of poorly bedded frozen muck that is ~10–35 m thick, contains abundant megafaunal bones, and on rare occasions, frozen partial carcasses[S11-S12]. It also contains abundant organic material including finely disseminated plant fragments and some layers of peat and larger plant debris[S2,S12], as well as the Chatanika Ash Bed (~14 ka B.P.) near the upper middle part of the formation[S2,S6]. The Goldstream Formation formed between ~125 and 10 ka B.P., is mostly Wisconsinan in age[S2,S7], and roughly correlates with the lower Silt unit or King Solomon Formation of the Klondike mucks[S5,S13]. There are several tephra beds within the King Solomon Formation including the Dawson tephra dated at ~25 ka B.P.[S5,S14].

The Ready Bullion Formation and laterally equivalent Engineer Loess (Fig. 2) unconformably overlie the Goldstream Formation. In creek-valley centers, the Ready Bullion Formation is ~1–10 m thick, thins upslope, and grades into the Engineer Loess, which varies in thickness between ~1 and 7 m[S2]. The Ready Bullion Formation is a frozen, poorly to well-stratified retransported loess overlying the Giddings Forest Bed (~10 ka B.P.)[S15]. The Engineer Loess contains upland silt of Holocene age, and both of these units are overlain by modern soils.

The Ready Bullion Formation additionally contains abundant and extremely well preserved plant material including logs and rooted stumps, branches and sticks, peat lenses, and carbonized plant fragments[S2]. Fewer vertebrate remains (including frozen carcasses) are found in the Ready Bullion Formation and are of Holocene animal forms that still live in the region[S12]. The upper Organic unit of the Klondike district (≤13 m thick) is equivalent to the Ready Bullion Formation and also consists of muck with abundant peat beds and plant macrofossils. The chaotic distribution of its enclosed logs and branches has been interpreted as representing the "occasional catastrophic accumulation" of these materials[S5].

Analytical methods

Scanning electron microscopy (SEM) and energy dispersive X-ray spectroscopy (EDS) techniques were used to distinguish between microspherules of impact origin and rounded detrital grains, authigenic framboids, or microspherules of volcanic, cosmic and anthropogenic origins[S16-S17]. Definitive identifications of impact-related microspherules can be made from SEM images showing dendritic-crystal (or skeletal) surface microstructures caused by high-temperature melting and quenching of target rock during impact cratering or atmosphere airbursts interacting with the ground surface[S16-S17]. In addition, EDS analyses were essential in providing the elemental composition data necessary to identify modes of formation for the candidate microspherules. Standard analytical techniques were used for EDS analyses, which were acquired multiple times and/or encompassed areas measuring ~50% of the diameter of each analysed microspherule, and calibrated using appropriate reference standards; the EDS compositions are considered to be precise within approximately ±5 wt.%[S18].

Accelerator mass spectrometer (AMS) radiocarbon dates for the fossil skull fragments, listed in Table S1, were determined on collagen extractions made at the commercial PaleoResearch Institute in Golden, Colorado. After surface cleaning the bone samples were decalcified with cold, dilute hydrochloric acid. Further processing was undertaken to remove particulate contamination, humates, fulvic acids, and any incidentally produced mineral salts. The purified collagen samples were sealed in quartz tubes, heated to convert the sample's carbon to CO_2 gas, and, along with a set of standards, blanks, and laboratory background samples, submitted to either the Keck Carbon Cycle AMS Facility at the University of California, Irvine, or the Center for Applied Isotope Studies at the University of Georgia, Athens. Following determination of ^{14}C to ^{12}C and ^{13}C ratios, the times elapsed since the animals' deaths were calculated as conventional radiocarbon ages and calibrated ages using the Intcalc04 curves in the program OxCal, version 3.10[S19-S20].

Bulk sediment samples of ~30–50 g each from four bison skulls and three primary loess localities (Table S1) were analysed for platinum (Pt) and palladium (Pd) abundances at Activation Laboratories Ltd., Ontario, Canada, using fire assay and inductively coupled plasma mass spectrometry (ICP-MS), following the methods of Hoffman and Dunn[S21]. Samples submitted from the fossil skulls were limited to those having enough original material remaining after the magnetic separates had been made. Accuracy of the laboratory results was verified using blanks and known standards, and the analyses have a lower detection limit of 0.1 parts per billion (ppb; Table S2).

Historical note (Figure 3 photos)

The photos in Figure 3 were taken when Frank C. Hibben, a University of New Mexico archeologist, and his wife Eleanor (aka Brownie) visited Alaska in the summer of 1941. They had come in search of evidence in support of the migration of North America's early human inhabitants across Beringia. As Eleanor recorded in her diary (courtesy of the Maxell Museum of Anthropology, University of New Mexico), the Hibbens visited Fairbanks to meet Otto Geist and see for themselves the megafaunal bones he was famous for collecting[S22]. On the first day, Sunday, August 3, they missed meeting up with Geist, but decided to drive out to the Cripple Creek mine without him. Eleanor wrote in her diary about the trip, "… we found 2 places where the bones were coming out of the muck, and collected several specimens of bison skulls, mammoth and mastodon remains, and various teeth. Also found some things in Geist's discard piles." One is presumably shown in Figure 3a.

On their second day in Fairbanks, August 4, they returned to Cripple Creek, this time accompanied by Geist (Fig. 3b), and, among other things, "examined the 4 ash layers of volcanic eruptions, the lower of which he [Geist] thinks eliminated the animals." Apparently, Geist had explained to them his ideas concerning the muck deposits' origins (see Introduction). Although Frank Hibben's subsequent publications (e.g., "The Lost Americans"[S23]) gave explanations of the mucks that closely resembled Otto Geist's views[S22], they were presented without attribution.

References

S1. Robinson, M. S., Smith, T. E. & Metz, P. A. Bedrock geology of the Fairbanks mining district. *AK Div. Geol. Geophys. Surv.* (1990).

S2. Péwé, T. L. Quaternary Stratigraphic Nomenclature in Central Alaska. *U.S. Geol. Surv. Prof. Pap. 862* (1975).

S3. Westgate, J. A., Stemper, B. A. & Péwé, T. L. A 3 m.y. record of Pliocene-Pleistocene loess in interior Alaska. *Geology*, **18**, 858-861 (1990).

S4. Péwé, T. L. *Geomorphology of the Fairbanks area, Alaska*. Ph.D. thesis (Stanford University, 1952).

S5. Fraser, T. A. & Burn, C. R. On the nature and origin of "muck" deposits in the Klondike area, Yukon Territory. *Can. J. Earth Sci*., **34**, 1333-1344 (1997).

S6. Péwé, T. L. Quaternary stratigraphy of the Fairbanks area, Alaska: *U.S. Geol. Surv. Circ. 1026*, 72-77 (1989).

S7. Péwé, T. L., Westgate, J. A., Preece, S. J., Brown, P. M. & Leavitt, S. W. Late Pleistocene Dawson Cut Forest Bed and new tephrochronological findings in the Gold Hill Loess, east-central Alaska. *Geol. Soc. Am. Bull*., **121**, 294-320 (2009).

S8. Muhs, D. R. *et al*. Stratigraphy and palaeoclimatic significance of late Quaternary loess-palaeosol sequences of the Last Interglacial–Glacial cycle in central Alaska. *Quat. Sci. Rev*., **22**, 1947–1986 (2003).

S9. Muhs, D. R. *et al*. Paleoclimatic significance of chemical weathering in loess-derived paleosols of subarctic central Alaska, *Arct., Antarct., Alp. Res*., **40**, 396-411 (2008).

S10. Berger, G. W. & Péwé, T. L. Last Interglacial age of the Eva Forest Bed, central Alaska, from thermoluminescence dating of bracketing loess. *Quat. Sci. Rev*., **20**, 485-498 (2001).

S11. Guthrie, R. D. *Frozen fauna of the mammoth steppe: The story of Blue Babe* (University of Chicago Press, 1990).

S12. Péwé, T. L. Quaternary Geology of Alaska. *U.S. Geol. Surv. Prof. Pap*., **835** (1975).

S13. Kotler, E. & Burn, C. R. Cryostratigraphy of the Klondike "muck" deposits, west-central Yukon Territory. *Can. J. Earth Sci*., **37**, 849–861 (2000).

S14. Froese, D. G. *et al*. The Klondike goldfields and Pleistocene environments of Beringia. *GSA Today*, **19**, 4-10 (2009).

S15. Péwé, T. L., Berger, G. W., Westgate, J. A., Brown, P. M. & Leavitt, S. W. Eva Interglaciation Forest Bed, Unglaciated East-Central Alaska: Global Warming 125,000 Years Ago. *Geol. Soc. Am. Spec. Pap. 319* (1997).

S16. Bunch, T. E. *et al*. Very high-temperature impact melt products as evidence for cosmic airbursts and impacts 12,900 years ago. *PNAS*, **109**, E1903-E1912 (2012).

S17. Wittke, J. H. *et al.* Evidence for deposition of 10 million tonnes of impact spherules across four continents 12,800 y ago. *PNAS*, **110**, E2088-E2097 (2013).

S18. Goldstein, J. I. *et al*. *Scanning Electron Microscopy and X-ray Microanalysis: A Text for Biologists, Materials Scientists, and Geologists* (Springer Science & Business Media, 2013).

S19. Ramsey, C. B. OxCal. 3.1 ed. http://www.rlaha.ox.ac.uk/oxcal/oxcal.htm (2005).

S20. Reimer, P. J. *et al*. IntCal09 and Marine09 radiocarbon age calibration curves, 0-50,000 years cal BP. *Radiocarbon*, **51**, 1111-1150 (2009).

S21. Hoffman, E. L. & Dunn, B. Sample preparation and bulk analytical methods for PGE in *The Geology, Geochemistry and Mineral Beneficiation of Platinum Group Elements* (ed. Cabri, L. J.) 1-11 (Canadian Institute of Mining, Metallurgy and Petroleum, 2002).

S22. Keim, C. J. *Aghvook, white eskimo: Otto Geist and Alaskan archaeology* (University of Alaska Press, 1969).

S23. Hibben, F. C. *The Lost Americans: The pre-Indian hunters whose bones, tools, and weapons reveal human life of 8,000-30,000 years ago* (Thomas Y. Crowell Company, 1946).

S24. Jensen, B. J. L., Evans, M. E., Froese, D. G. & Kravchinsky, V. A. 150,000 years of loess accumulation in central Alaska. *Quat. Sci. Rev.*, **135**, 1-23 (2016).

TABLE S1. "MUCK" SEDIMENT SAMPLES, MAGNETIC SEPARATES, AND MICROSPHERULES FROM ALASKAN AND YUKON PLEISTOCENE MEGAFAUNAL FOSSILS AND ALASKAN PRIMARY LOESS SECTIONS

Source[1]	Source ID[1]	Location	Age[2]	Sediment[3] sample	Magnetic[4] separates	Magnetic[5] spherules	Carbonate[6] spherules
Bison skull	A-501-1027	Fairbanks, AK	18,635 ± 65	45.20*	1.94	16,970	0
Bison skull	Y-301.16	Klondike, YT	29,065 ± 105	26.00	0.13	3210	0
Rangifer skull	Y-109.8	Klondike, YT	30,125 ± 275	63.40	1.06	0	0
Bison skull	Y-3.9	Klondike, YT	37,000 ± 185	30.40	1.35	1050	0
Bison skull	Y-406.1	Klondike, YT	40,155 ± 245	60.00*	2.15	1950	0
Bison skull	Y-3.28	Klondike, YT	40,950 ± 1500	83.50*	0.77	6230	140
Mammoth skull	A-478-3499	Fairbanks, AK	48,200 ± 2750	64.80	1.05	17,800	0
Bison skull	A-698-2022/23	Fairbanks, AK	*undated*	24.70	0.70	9720	0
Bison skull	Y-404-716	Klondike, YT	*undated*	32.11*	0.42	0	0
Primary loess	AK-211	Halfway House	~9,900	61.84*	9.72	0	0
Primary loess	AK-213	Halfway House	~11,600	62.84	4.92	0	0
Primary loess	AK-225	Halfway House	~54,200	58.96*	3.47	0	0
Primary loess	AK-906A	Chena Hot Springs Road	~9,400	63.30*	1.88	0	0
Primary loess	AK-915	Chena Hot Springs Road	~36,200	60.50	1.49	17(?)	0
Primary loess	AK-924	Chena Hot Springs Road	~40,800	60.54	3.38	0	0
Primary loess	AK-720	Birch Hill	~34,000	51.91	1.08	0	0
Primary loess	AK-728	Birch Hill	~41,500	60.60	1.54	0	0

[1]Alaskan (AK) fossils from the American Museum of Natural History collection (A-) in New York City, and Yukon Territory (YT) fossils from the Territorial Government collection (Y-) in Whitehorse. Alaskan and Yukon samples are presumably from the Goldstream and King Solomon Formations, respectively. The Alaskan primary loess samples (AK-) were collected by D. Muhs (USGS) at the sections indicated in the lower half of the table. These primary loess samples are equivalent in age to the mucks, but do not appear in T. Péwé's stratigraphic classification (Fig. 2; Supplementary Information, "Quaternary stratigraphy").

[2]CAL yr B.P. (1σ) bone collagen dates determined by the PaleoResearch Institute, Golden, CO; loess sample age estimates (CAL yr B.P.) are based on radiocarbon and other dates listed in Muhs et al.[S8,S9] and Jensen et al.[S24] (see text)

[3]Weight of sediment samples (g); *sediment sample submitted for platinum and palladium analyses (see Table S2)

[4]Weight of magnetic separates (g)

[5]Number of magnetic microspherules/kg; only one spherical particle of unknown origin (~15-micron diameter) was found in sample AK-915, which is equivalent to ~17 microspherules/kg

[6]Number of carbonate microspherules/kg

TABLE S2. PLATINUM AND PALLADIUM ANALYSES FOR SEDIMENT FROM ALASKAN AND YUKON PLEISTOCENE FOSSILS AND ALASKAN PRIMARY LOESS SECTIONS

Source[1]	Source ID[1]	Location	Pt[2]	Pd[3]	Pt/Pd
Bison skull	A-501-1027	Fairbanks, AK	3.9	1.0	3.9
Bison skull	Y-406.1	Klondike, YT	3.1	0.9	3.4
Bison skull	Y-3.28	Klondike, YT	2.2	1.2	1.8
Bison skull	Y-404-716	Klondike, YT	0.9	1.1	0.8
Primary loess	AK-211	Halfway House	1.3	1.9	0.7
Primary loess	AK-225	Halfway House	1.1	1.5	0.7
Primary loess	AK-906A	Chena Hot Springs Road	0.8	1.6	0.5

[1]Alaskan (A-, AK) and Yukon Territory (Y-, YT) fossil sources, IDs, and locations as given in Table S1
[2]Platinum (Pt) abundances in parts per billion (ppb) with a lower detection limit of 0.1 ppb
[3]Palladium (Pd) abundances in ppb with a lower detection limit of 0.1 ppb

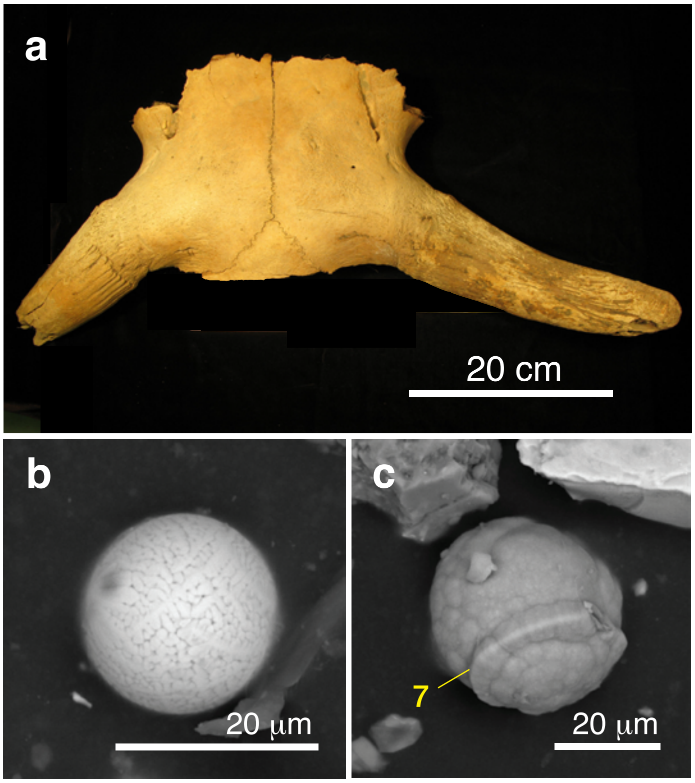

Figure S1. (a) Photograph of Yukon bison skull (Y-406.1; *Bison priscus*) from the Yukon Territorial Government collection in Whitehorse recovered at Lower Quartz Creek on 24 July 2011. The skull has been dated at 40,155 ± 245 CAL yr B.P. (Table S1). (b-c) SEM images of two microspherules magnetically separated from fine-grained sediment contained within the skull. Yellow number 7 indicates an apparent aerodynamic flange.

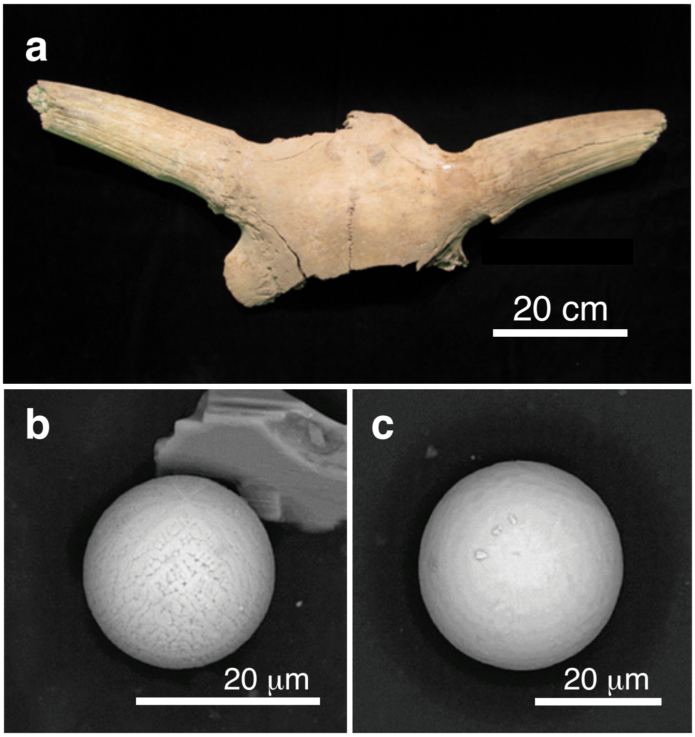

Figure S2. (a) Photograph of Yukon bison skull Y-3.9 (*Bison priscus*) from the Territorial Government collection recovered at Lower Quartz Creek in the Klondike district. The skull has been dated at 37,000 ± 185 CAL yr B.P. (Table S1). (b-c) SEM images of two microspherules magnetically separated from fine-grained sediment contained within the skull.

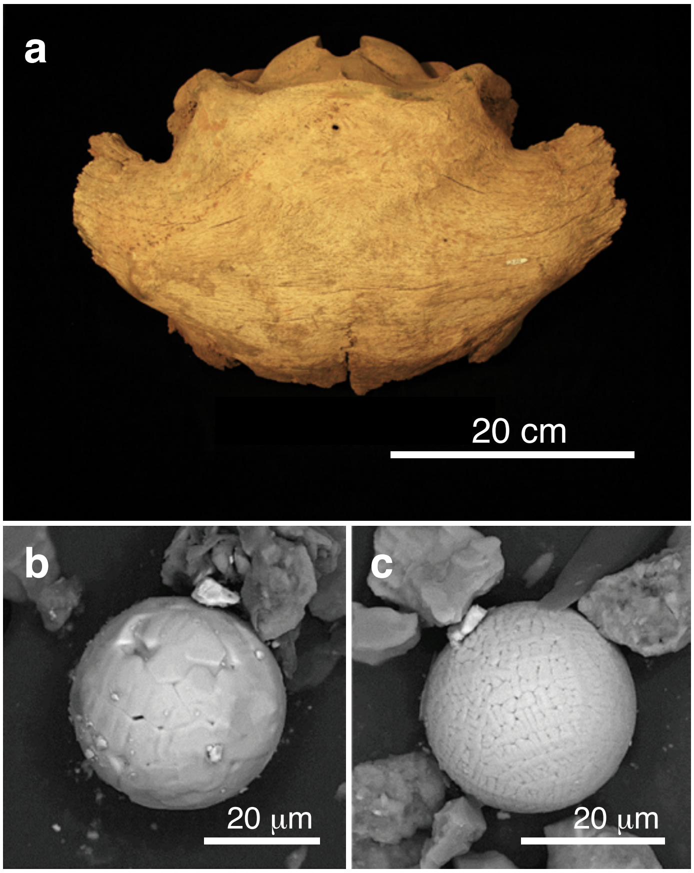

Figure S3. (a) Photograph of Yukon bison skull Y-3.28 (*Bison priscus*) from the Territorial Government collection recovered in the Klondike district. The skull has been dated at 40,950 ± 500 CAL yr B.P. (Table S1). (b-c) SEM images of two microspherules magnetically separated from fine-grained sediment contained within the skull.

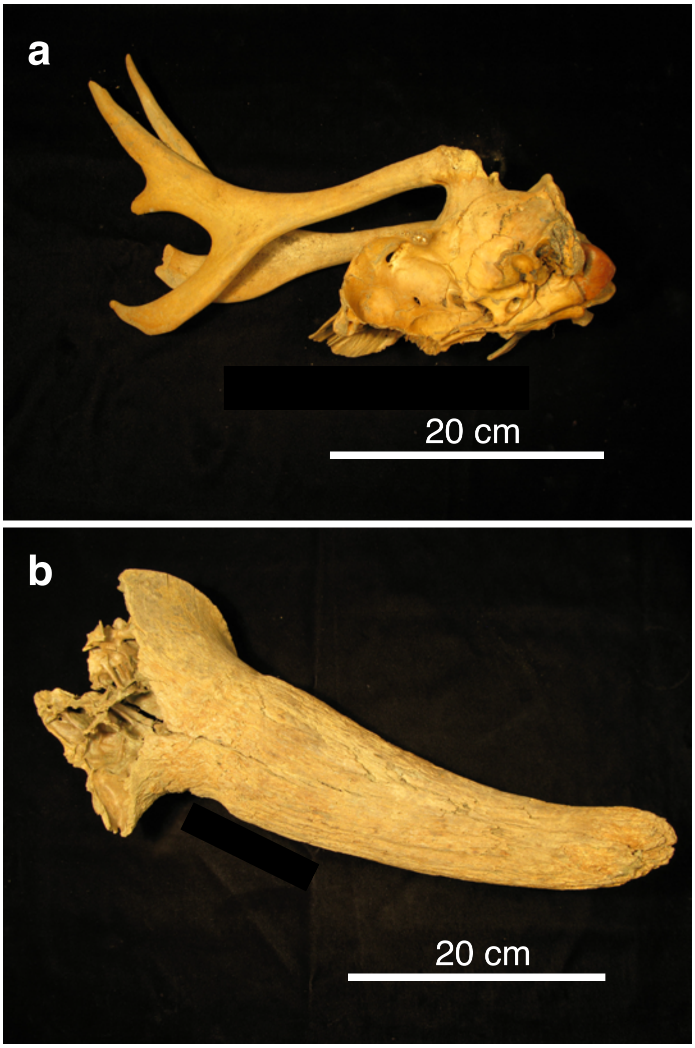

Figure S4. Photographs of (a) Rangifer Y-109.8 and (b) Yukon bison Y-404.716 (*Bison priscus*) skulls from the Territorial Government collection recovered in the Klondike district that contained sediment but no magnetic microspherules (Table S1). Notably, the rangifer skull is the least damaged of the skulls collected for this study.

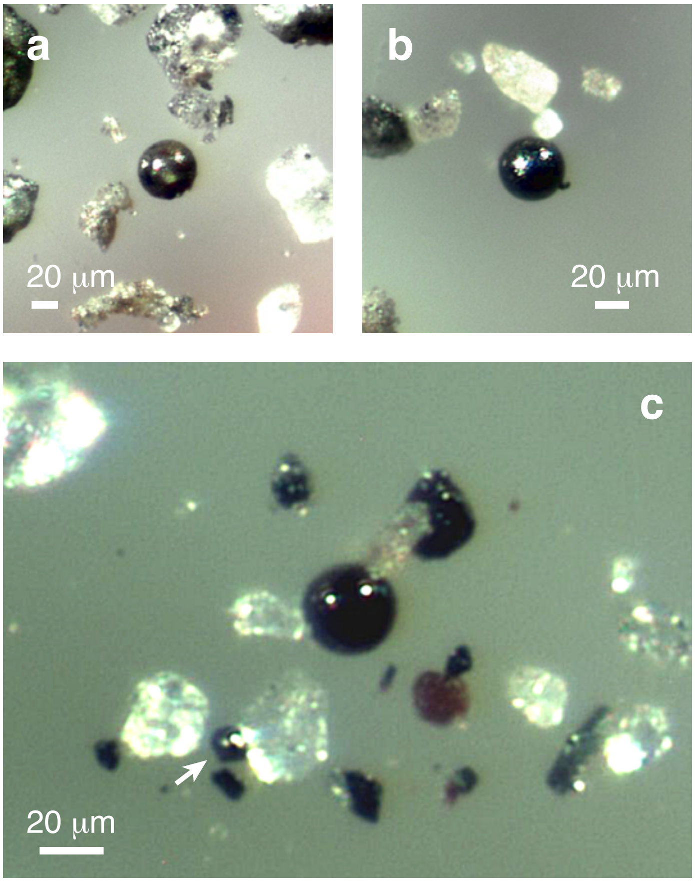

Figure S5. Microspherules under reflected light (a, b) from bison skull A-501-1027 (Fig. 4); and (c) from mammoth skull A-478-3499 (Fig. 5); note smaller microspherule at lower left (white arrow).
